# Polyclonal T-Cells Express CD1a in Langerhans Cell Histiocytosis (LCH) Lesions

**DOI:** 10.1371/journal.pone.0109586

**Published:** 2014-10-24

**Authors:** Jennifer A. West, Sharon L. Olsen, Jenée M. Mitchell, Ross E. Priddle, Jennifer M. Luke, Selma Olsson Åkefeldt, Jan-Inge Henter, Christopher Turville, George Kannourakis

**Affiliations:** 1 Fiona Elsey Cancer Research Institute, Ballarat, Victoria, Australia; 2 School of Health Sciences, Federation University, Mt Helen, Victoria, Australia; 3 Childhood Cancer Research Unit, Department of Women’s and Children’s Health, Karolinska Institutet, Karolinska University Hospital, Stockholm, Sweden; 4 School of Science, Information Technology and Engineering, Federation University, Mt Helen, Victoria, Australia; INSERM, France

## Abstract

Langerhans cell histiocytosis (LCH) is a complex and poorly understood disorder that has characteristics of both inflammatory and neoplastic disease. By using eight-colour flow cytometry, we have identified a previously unreported population of CD1a^+^/CD3^+^ T-cells in LCH lesions. The expression of CD1a is regarded as a hallmark of this disease; however, it has always been presumed that it was only expressed by pathogenic Langerhans cells (LCs). We have now detected CD1a expression by a range of T-cell subsets within all of the LCH lesions that were examined, establishing that CD1a expression in these lesions is no longer restricted to pathogenic LCs. The presence of CD1a^+^ T-cells in all of the LCH lesions that we have studied to date warrants further investigation into their biological function to determine whether these cells are important in the pathogenesis of LCH.

## Introduction

Langerhans cell histiocytosis (LCH) is a complex disease with unpredictable progression and no known cause [1], [2]. LCH occurs predominantly in children but also occurs in adults. Lesions are most common in bone (eosinophilic granuloma) and skin, but may occur in other organs. LCH may be confined to single sites, have multifocal involvement or become disseminated. The clinical course varies from lesions that spontaneously resolve, to a chronic disease, or can be disseminated and life-threatening [1]. The severity and prognosis are dependent on the type and extent of organ involvement, with children under two most at risk of life-threatening complications.

LCH was originally defined by the Writing Group of the Histiocyte Society in 1987 [3], and was more recently revised [4]. LCH lesions are diagnosed by an accumulation of cells, long presumed to be pathogenic Langerhans cells (LCs) [5], [6]. The standard identification of LCs is by their morphology (including Birbeck granules) or with the immunological marker CD1a [7], [8]. Langerin (CD207) has also been used as a marker of LCs [9]. Other inflammatory cells typically found in LCH lesions include T-cells, eosinophils, plasma cells, neutrophils, basophils, macrophages and giant cells [10].

The recognition of close similarities between normal epidermal LCs and pathogenic LCs has led to the concept that epidermal LCs are the precursor cells in LCH [5]. LCH combines in one nosological category, a group of disorders that have differing clinical manifestations, but all are characterized by an accumulation of cells with features of cutaneous LCs and inflammatory cells. The presence of inflammatory cells in all LCH lesions implies that a better understanding of these cells may lead to improvements in the management of this group of disorders.

Most LCH research has focused on pathogenic LCs. While the accumulation of pathogenic LCs within LCH lesions is considered to be a defining characteristic of LCH [3], their role in the causation or impact of the disease remains unclear. LCH has pathological features of both cancer and chronic inflammation, and whether it is a true malignancy remains contentious [11]–[13]. The CD1a-expressing cells have been reported to be clonal and pathogenic [14], [15], and this is supported by the detection of *BRAF* mutations in LCH lesions [16], [17].

The unresolved role of T-cells in LCH is indicated by the number of conflicting reports relating to the types of T-cells within lesions [6], [18]–[22]. Debate also surrounds the detection of high serum levels of IL-17A during active LCH and IL-17A synthesis by dendritic cells (DCs) in LCH lesions [20]. Subsequent studies did not support these findings [21], [22]. Expansion of regulatory T-cells has been associated with the accumulation of LCs in LCH lesions [6], [18], although more research is warranted to elucidate the role of T-cells in these lesions.

CD1a expression on pathogenic Langerhans cells (LCs) in LCH is regarded as a hallmark of this disease, but the role of this highly restricted molecule in LCH has remained uncertain. Normally, CD1a molecules on the surface of LCs present glycolipid antigens to specialized T-cells as part of their role in immunosurveillance [23], [24]. Stimuli from pathogens, tumors or host immune responses that are capable of modifying CD1a expression have demonstrated similar results *in vitro* [23]. Expression of CD1a is thought to be partly controlled by cytokines, as demonstrated by the induction of CD1a in cultured myeloid progenitor cells [25],[26].

While CD1a is expressed predominantly on LCs, it can be expressed by T-cells under certain conditions. Immature thymocytes express CD1a [27], as do some T-cells involved in neoplastic conditions such as pre-lymphoblastic lymphomas/leukemias [28]–[31] and follicular dendritic cell sarcoma [32]. T-cells that express CD1a have recently been identified within the tonsil suggesting extrathymic T-cell development [33]. To date, there are no reports of CD1a expression on mature T-cells.

Historically, the cellular composition of LCH was determined using archival tissue sections. This approach formed the foundation of what is currently known about this disease, however, these earlier studies were greatly restricted by the number of CD markers that could be detected simultaneously. LCH tissues have been examined by flow cytometry in a limited number of studies [34], [35] and in some cases, specific cell populations have been sorted for further analysis [6], [36]. These studies confirmed the presence of pathogenic LCs using CD1a and/or CD207 [34]. Sorted CD1a^+^ cells from LCH lesions expressed CD207 in more than 75% of the cells, and these were deficient in stimulating allogeneic T-cell proliferation *in vitro* [34].

We set out to better define the role of CD1a and T-cells in LCH lesions, using multi-parameter flow cytometry with up to eight cell markers simultaneously. Here we report for the first time, to our knowledge, the expression of CD1a on polyclonal T-cells in LCH lesions. The results provide new insights into the composition of LCH lesions and we anticipate that future studies into the biological function of these cells may lead to an improved understanding of LCH pathogenesis.

## Results

### Identification of CD1a^+^/CD3^+^ cells by flow cytometry

**Figure 1:**
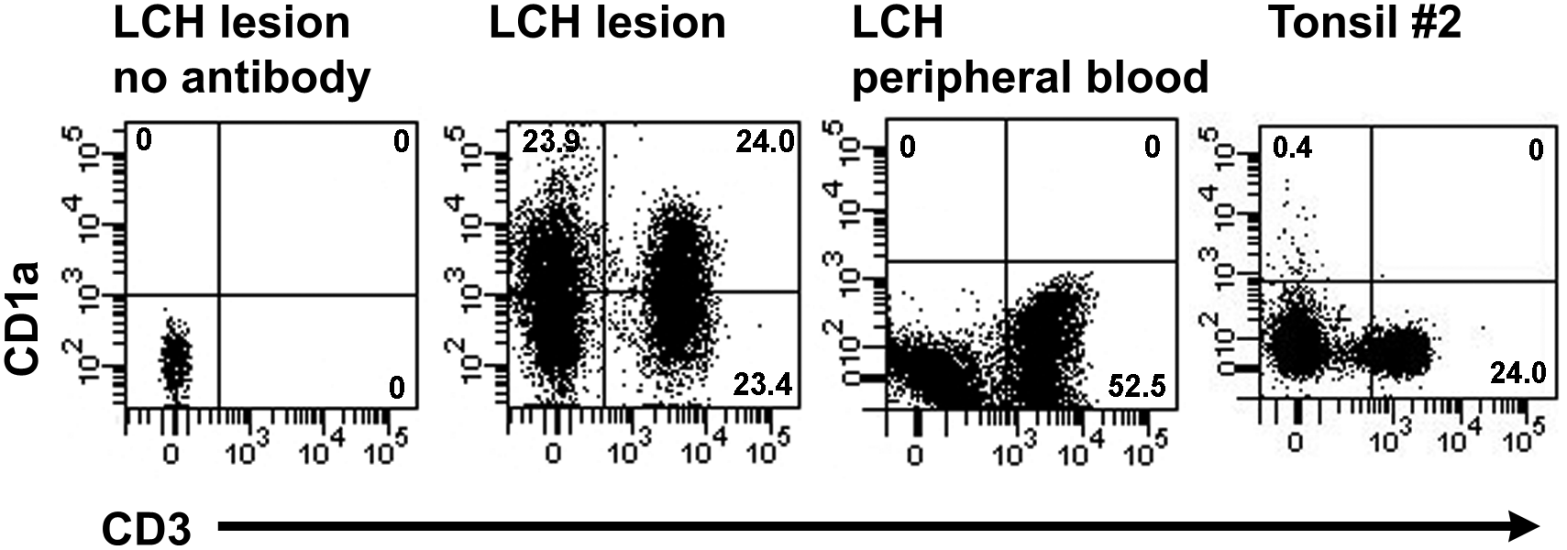
Identification of CD1a^+^/CD3^+^ cells from LCH lesions by flow cytometry. Representative plots showing anti-CD1a-FITC [NA 1/34] and anti-CD3-APC-H7 [SK7] staining of lesional cells and peripheral blood from LCH patient #1, and cells extracted from control Tonsil #2. The unstained (left) plot shows approximately 500 live cells. Remaining plots display 30,000 live cells or greater. Live cells are defined as those cells remaining after doublet and propidium iodide positive cells were excluded. The quadrant numbers indicate the percentage of each population within the live cell gate.

Using flow cytometry we characterized the cell marker profiles of LCH lesions from six patients (Table 1, 2). Within the LCH lesion from patient #1, we identified an unexpected population of CD1a^+^/CD3^+^ cells that constituted 23.8% of the live-gated cells (Table 3). A representative plot of these CD1a^+^/CD3^+^ cells from a single experiment is shown in Figure 1. Although the CD1a^+^ population represented 44.7% of the cells in this LCH sample, over half of the CD1a^+^ cells (53.2%) also co-expressed CD3 (Table 3). This represented 56.7% of the total CD3^+^ cells. These CD1a^+^/CD3^+^ cells were absent in the peripheral blood of this patient and a tonsil control tissue (Figure 1). CD1a^+^/CD3^+^ cells were subsequently identified in a further five LCH patients (Table 3).

**Table 1:** Clinical details of LCH patients.

Patient	Age	Gender	LCH diagnosis	Tissue examined
#1	9	M	Eosinophilic granuloma of skull and lymph nodelesion	Lesion Peripheral blood
#2	12	F	Multifocal eosinophilic granulomas of skull andlymph node lesions	Lesion
#3	58	F	Multifocal dermal lesions of shin	Lesion Peripheral blood
#4	6	F	Single eosinophilic granuloma of skull	Lesion Peripheral blood
#5	3	M	Multifocal eosinophilic granulomas (right tibia,parietal bone, costae)	Lesion Peripheral blood
#6	13	F	Single eosinophilic granuloma of skull	Lesion
#7	31	M	Disseminated	Peripheral blood
#8	53	M	Multifocal (bone, lung)	Peripheral blood
#9	61	M	Multifocal (bone, lung)	Peripheral blood
#10	1	M	Disseminated	Peripheral blood
#11	2	F	Disseminated	Peripheral blood

**Table 2:** Experimental details for LCH patients.

Patient	Tissueexamined	No. ofexperimentsperformed	Total no. of livecells examined	CD markers examined by flow cytometry
#1	Lesion	5	72,409	1a, 3, 4, 8, 14, 16, 19, 25, 31, 34, 45, 45RA, 45RO, 56, 123, 138, 207, TCR Vβ
	PeripheralBlood	1	40,523	1a, 3, TCR Vβ
#2	Lesion	1	145,329	1a, 3
#3	Lesion	3	9,150	1a, 3, 4, 8, 14, 19, 34, 45, 45RA, 45RO, 123, 138, TCR Vβ
	PeripheralBlood	1	9,743	1a, 3, TCR Vβ
#4	Lesion	5	105,617	1a, 3, 4, 8, 14, 16, 19, 25, 31, 34, 45, 45RA, 45RO, 56, 123, 138, 207, TCR Vβ
	PeripheralBlood	1	9,140	1a, 3
#5	Lesion	1	2,002	1a, 3, 4, 8, 19, 45
	PeripheralBlood	1	10,835	1a, 3, TCR Vβ
#6	Lesion	4	25,586	1a, 3, 4, 8, 14, 19, 34, 45, 45RA, 45RO, 123, 138
#7	PeripheralBlood	1	42,437	1a, 3, TCR Vβ
#8	PeripheralBlood	1	22,043	1a, 3, 8, 14, 25, 123, 207, TCR Vβ
#9	PeripheralBlood	1	60,319	1a, 3, 4, 16, 19, 31, 56, TCR Vβ
#10	PeripheralBlood	1	202,873	1a, 3
#11	PeripheralBlood	1	65,273	1a, 3, 4, 16, 19, 31, 56

**Table 3:** The mean percentages of CD1a^+^ cells, CD1a^+^/CD3^+^ cells and CD3^+^ cells in the live cell population of lesional cells from six LCH patients.

LCH Patient	% CD1a^+^	% CD1a^+^/CD3^+^	% CD3^+^
**#1**	44.7	23.8	42.0
**#2**	3.9	1.5	5.3
**#3**	6.6	5.3	29.3
**#4**	22.5	2.3	9.4
**#5**	6.5	3.0	43.9
**#6**	31.0	13.6	45.2

CD1a^+^/CD3^+^ cells were not detected in normal peripheral blood from six volunteers, in peripheral blood from nine LCH patients or in single cell suspensions (SCS) prepared from the epithelial layer of five tonsils (Table 2, 4). Two acute myeloid leukemia (AML) samples, one chronic lymphoid leukemia and one normal lymph node were profiled and no CD1a^+^/CD3^+^ cells were identified. A Kruskal-Wallis test was conducted to compare the mean ranks of the percentage of CD1a^+^/CD3^+^ cells for LCH lesions (*n* = 6, 8.3±8.8), LCH peripheral blood tissues (*n* = 9, 0.0±0.0), and control tissues (*n* = 15, 0.0±0.0). There was a significant difference across the three groups (H(2) = 28.54, p<0.0001) with a mean rank of 27.5 for LCH lesions and 12.5 for the other groups. Dunn’s multiple comparisons test revealed significant differences between LCH lesions and LCH peripheral blood tissues (p<0.0001) and LCH lesions and control tissues (p<0.0001). One T-cell lymphoma contained 0.4% CD1a^+^/CD3^+^ cells. This was not unexpected, as immature T-cells have been reported to express both CD1a and CD3 [32].

**Table 4:** Experimental details for control patients.

Tissue anddescription	No. of experimentsperformed	Total no. ofcells examined	CD markers examined by flowcytometry
Tonsil #1	1	7,812	1a, 3, 4, 16, 19, 31, 56
Tonsil #2	1	21,581	1a, 3, 4, 16, 19, 31, 56
Tonsil #3	1	9,134	1a, 3, 4, 45RA, 45RO
Tonsil #4	1	11,072	1a, 3, 8, 14, 25, 123, 207
Tonsil #5	1	10,417	1a, 3, 8, 14, 25, 123, 207
Lymph node (LCHpatient with noinvolvement atthis site)	1	6,255	1a, 3, 4, 16, 19, 31, 56
Peripheral Blood 1	1	14,989	1a, 3, 4, 16, 19, 31, 56
Peripheral Blood 2	3	21,468	1a, 3, 4, 8, 14, 16, 19, 25, 31, 45, 56, 123, 207
Peripheral Blood 3	2	7,103	1a, 3, 4, 8, 14, 19, 34, 45, 123, 138
Peripheral Blood 4	1	2,776	1a, 3, 4, 8, 19, 45
Peripheral Blood 5	1	1,648	1a, 3, 4, 8, 19, 45
Peripheral Blood 6	2	15,101	1a, 3, 4, 8, 14, 19, 34, 45, 123, 138
Peripheral Blood 7(AML)	1	10,966	1a, 3, 4, 16, 19, 31, 56
Peripheral Blood 8(AML)	2	1,767	1a, 3, 4, 8, 14, 16, 19, 25, 31, 56, 123, 207
Peripheral Blood 9(CLL)	1	94,100	1a, 3, 4, 16, 19, 31, 56
Peripheral Blood 10(T-cell lymphoma)	1	248,717	1a, 3, 4, 45RA, 45RO

### CD1a^+^/CD3^+^ cells are morphologically and phenotypically polyclonal T-cells

**Figure 2:**
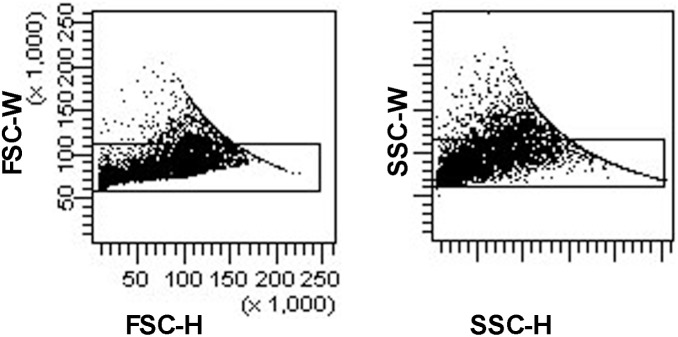
Doublet gate plots. Representative plots from LCH patient #1 showing the gates used to exclude cell doublets.

**Figure 3:**
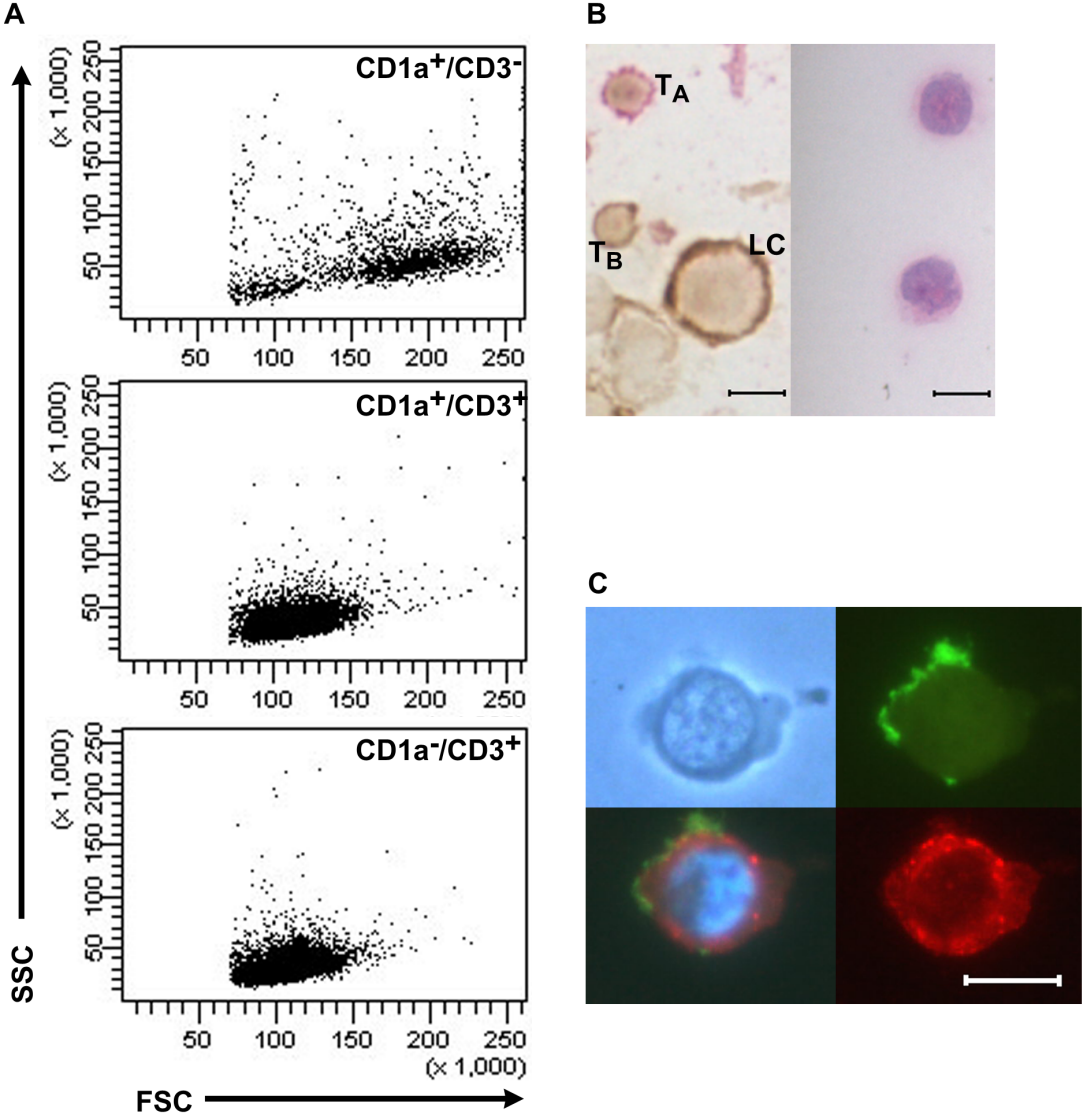
CD1a^+^/CD3^+^ cells from LCH lesions are morphologically and phenotypically polyclonal T-cells. (A) Relative size of CD1a^+^/CD3^−^ LCs (mean forward scatter intensity = 1.7×10^5^), CD1a^+^/CD3^+^ T-cells (1.1×10^5^) and CD1a^−/^CD3^+^ T-cells (1.1×10^5^) on flow cytometric profiles from patient #1 (B-cells were excluded). Plots show staining for anti-CD1a-FITC [NA 1/34] and anti-CD3-APC-H7 [SK7]. (B) (Left) Immunocytochemical staining of a cytospin from a single cell suspension prepared from the lesion of patient #1 using anti-CD1a (DAB+/brown) and anti-CD3 (Fast Red), counterstained with Mayer’s hematoxylin and mounted in Dako Ultramount (Scale bar = 5 µm). Image shows a typical lymphocyte (T_A_), a T-cell with CD1a staining (T_B_), and a Langerhans cell (LC). (Right) H&E stain showing typical lymphocyte morphology of FACS-sorted CD1a^+^/CD3^+^ cells (Scale bar = 5 µm). (C) Phase contrast image (top left) and fluorescent images (Scale bar = 5 µm). Double immunofluorescence labeling of a CD1a^+^/CD3^+^ T-cell from a single cell suspension prepared from the lesion of patient #1, using anti-CD1a-AlexaFluor 488 (green), anti-CD3-AlexaFluor 594 (red). The nucleus is stained with DAPI (blue). The filter used for the bottom left image allows simultaneous viewing of all colors. Microscopy was performed on a Leica DMLB microscope (Leica Microsystems). Images were captured with a Leica DC300F digital camera (Leica Microsystems).

Tight doublet gates were applied to ensure that CD1a^+^/CD3^+^ cells were not two adjoined cells (Figure 2). No doublets were observed when sorted cells were examined under the microscope. In flow cytometry plots, the relative size and mean forward scatter intensity of the CD1a^+^/CD3^+^ cells was more typical of T-cells than LCs (Figure 3A). Similarly, the morphology of the sorted CD1a^+^/CD3^+^ cells, as seen microscopically, was more typical of lymphocytes than LCs (Figure 3B). When fluorescent immunocytochemistry was performed on lesional cells, some individual cells clearly expressed both CD1a and CD3 (Figure 3C).

### Further phenotyping of CD1a^+^ T-cells

Since the morphology of CD1a^+^/CD3^+^ cells was more typical of lymphocytes than LCs, further experiments were performed to determine their phenotype. The T-cell receptor (TCR) Vβ repertoires of the T-cells within lesions from three LCH patients were examined. Most of the 25 TCR Vβ subsets were detected in lesional CD1a^+^ T-cells without any obvious bias (data not shown). There was insufficient sample to analyze the remaining three LCH lesions. Because there were multiple TCR subtypes, CD1a^+^ T-cells could not have arisen from a single parent cell, and were therefore not clonal in origin.

**Figure 4:**
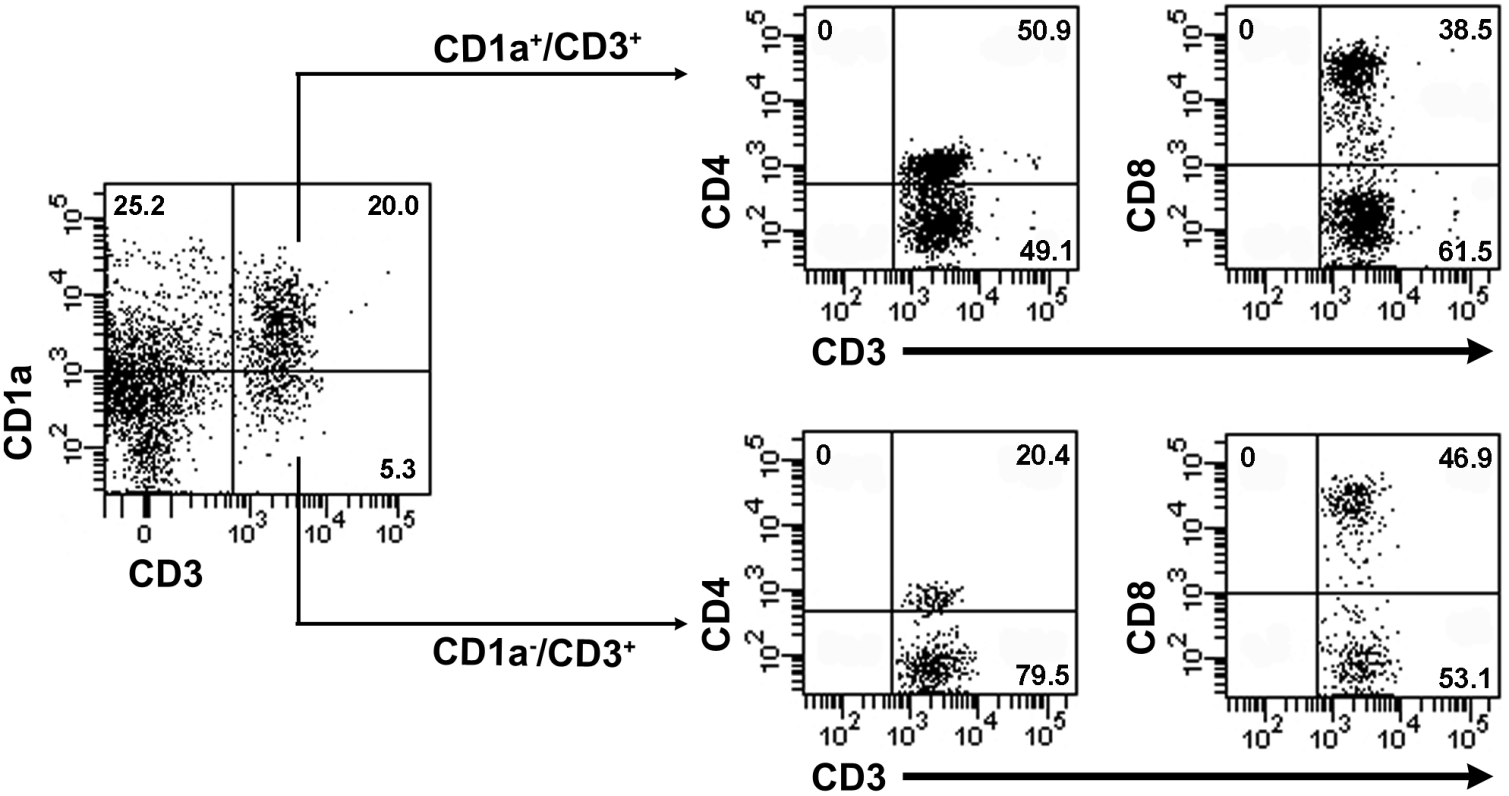
CD1a expression on T-cells is not restricted to either CD4^+^ or CD8^+^ subsets. The flow cytometry plots are from one experiment using lesional cells from LCH patient #1. Live cells were gated into quadrants based upon CD1a and CD3 antibody intensity and the percentages of cells were calculated. Live cells are defined as those cells remaining after doublet and propidium iodide positive cells were excluded. CD1a^+^/CD3^+^ cells were then gated to identify CD1a^+^/CD3^+^/CD4^+^ cells and CD1a^+^/CD3^+^/CD8^+^ cells. Similarly, CD1a^−/^CD3^+^ cells were gated to identify CD1a^−/^CD3^+^/CD4^+^ cells and CD1a^−/^CD3^+^/CD8^+^ cells. Plots show staining for anti-CD1a-APC [HI149], anti-CD3-APC-H7 [SK7], anti-CD4-V450 [RPA-T4] and anti-CD8-PE [HIT8a].

The T-cell subsets within five LCH lesional samples were examined to differentiate between CD4^+^ helper (T_H_) T-cells and CD8^+^ cytotoxic (T_C_) T-cells. One sample was excluded due to insufficient data. CD1a expression on CD1a^+^/CD3^+^ cells was not restricted to T_H_ or T_C_ subsets (Figure 4, Table 5). For LCH lesions, Mann-Whitney tests were conducted to compare the median percentage of CD1a^+^ T_H_ cells (*n* = 5, 60.1±26.6) with CD1a^−^ T_H_ cells (n = 5, 50.2±17.9), and the percentage of CD1a^+^ T_C_ cells (n = 5, 12.9±11.4) with CD1a^−^ T_C_ cells (n = 5, 19.5±10.6). No statistically significant differences were detected amongst the CD1a^+^ and the CD1a^−^ T_H_ groups (*U* = 9, p = 0.53) or the CD1a^+^ and the CD1a^−^ T_C_ groups (*U* = 7, p = 0.31). These results indicate that the proportions of T_H_ and T_C_ cells are not unusual in the CD1a^+^ T-cells when compared to other (CD1a^−^) T-cells.

**Table 5:** CD1a expression is not restricted to CD4^+^ or CD8^+^ T-cell subsets.

		LCH Patient			
% of T-cells		#1	#4	#5	#6
**CD1a^+^**	**CD3^+^**	56.7	24.4	6.8	30.2
	**CD3^+^/CD4^+^**	32.1	17.4	5.1	23.1
	**CD3^+/^CD8^+^**	18.4	2.2	0.5	8.5
**CD1a^−^**	**CD3^+^**	43.3	80.2	93.2	70.6
	**CD3^+^/CD4^+^**	14.1	30.7	71.6	40.4
	**CD3^+^/CD8^+^**	13.6	10.2	15.5	11.0

Mean data are expressed as percentages of T-cells from four LCH samples.

Additional CD markers were used where possible, to further phenotype the CD1a^+^ T-cells (Table 2, 6). As expected, these cells co-expressed the common leukocyte marker CD45. There was also variable, but low expression of T-cell markers including CD25 (expressed on T-cells during activation), and CD16 and CD56 (found on NKT-cells). Whilst the CD1a^+^ T-cells displayed some typical T-cell markers, most of the non T-cell markers examined were absent, or, present at negligible levels. These included CD19 (a B-cell marker), CD138 (a plasma cell marker), CD14 (a monocyte/macrophage marker) and CD34 (found on endothelial cells, DCs and progenitor cells). CD123 is not expressed on T-cells and was absent on the CD1a^+^ T-cells. Low levels of CD207 expression were detected in the two LCH samples examined (Table 6).

**Table 6:** Percentage of CD1a^+^ T-cells that express additional CD markers.

Additional CD marker	Patient number			
	#1	#4	#5	#6
CD207	26.8	11.7	ND*	ND
CD45	100.0	98.4	93.3	87.4
CD16	8.5	51.4	ND	ND
CD25	4.1	41.5	ND	ND
CD56	1.4	10.8	ND	ND
CD14	3.1	7.4	ND	4.8
CD34	0.0	4.1	ND	1.1
CD123	0.1	3.3	ND	0.3
CD138	0.0	4.9	ND	0.9
CD19	1.0	1.9	3.3	0.6

*ND (not determined).

**Figure 5:**
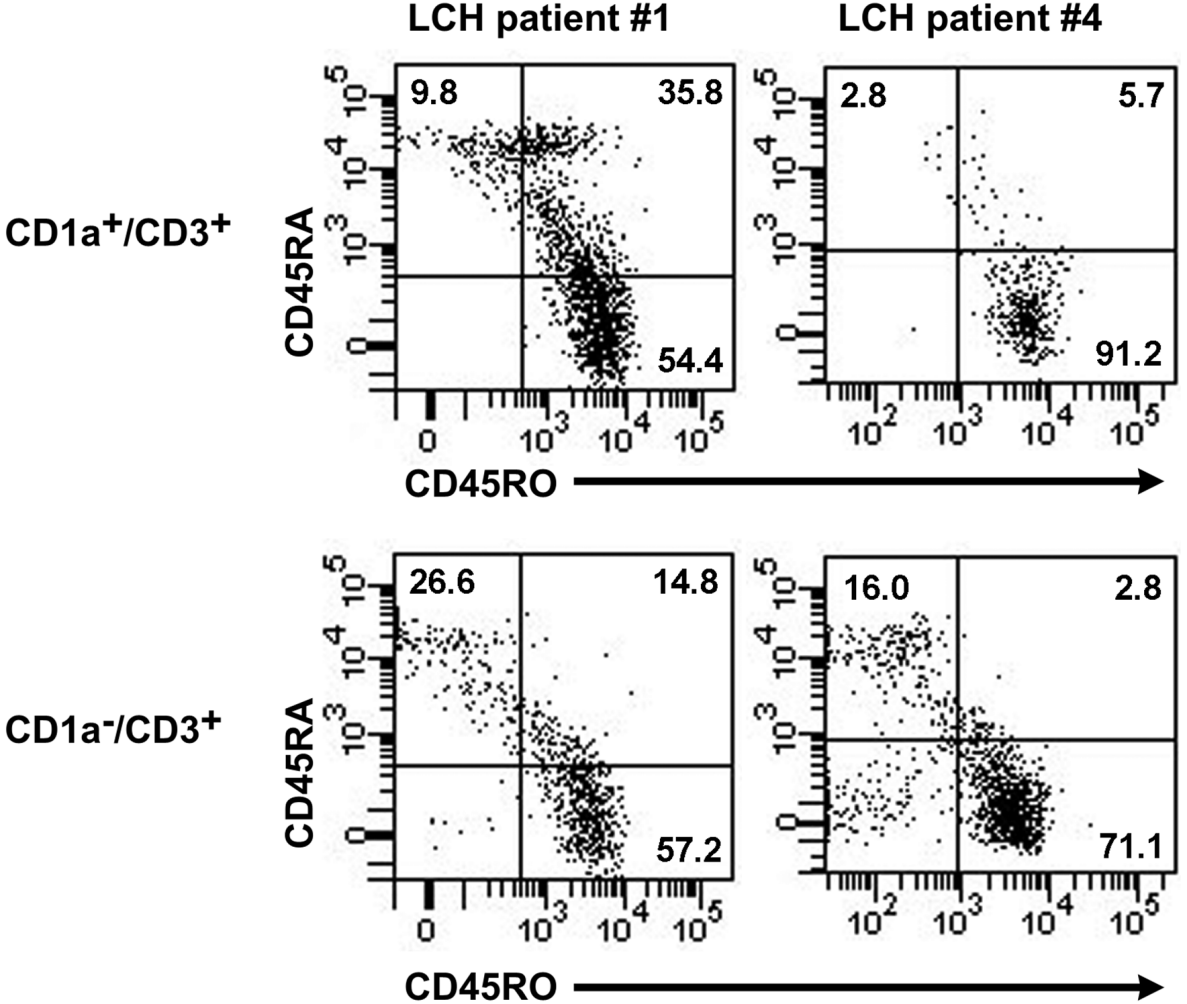
CD1a^+^ T-cells are not restricted to either naïve or memory T-cells. Expression of the T-cell activation markers CD45RA and CD45RO in CD1a^+^ and CD1a^−^ T-cells in flow cytometry plots of lesional cells from LCH patients #1 and #4. Plots show staining for anti-CD1a-FITC [NA 1/34], anti-CD3-APC-H7 [SK7], anti-CD45RA-PE-Cy7 [HI100] and anti-CD45RO-PE [UHCL-1].

It is known that upon recognition of specific antigen, naïve T-cells (CD45RA^+^/CD45RO^−^) reduce their expression of CD45RA and increase their expression of CD45RO, producing memory T-cells (CD45RA^−/^CD45RO^+^) [37]. We looked at CD45RA/CD45RO co-expression on CD1a^+^ T-cells and our results show that CD1a expression is not restricted to either naïve or memory T-cells (Figure 5).

### Sorted CD1a^+^ T-cells express CD1a mRNA

**Figure 6:**
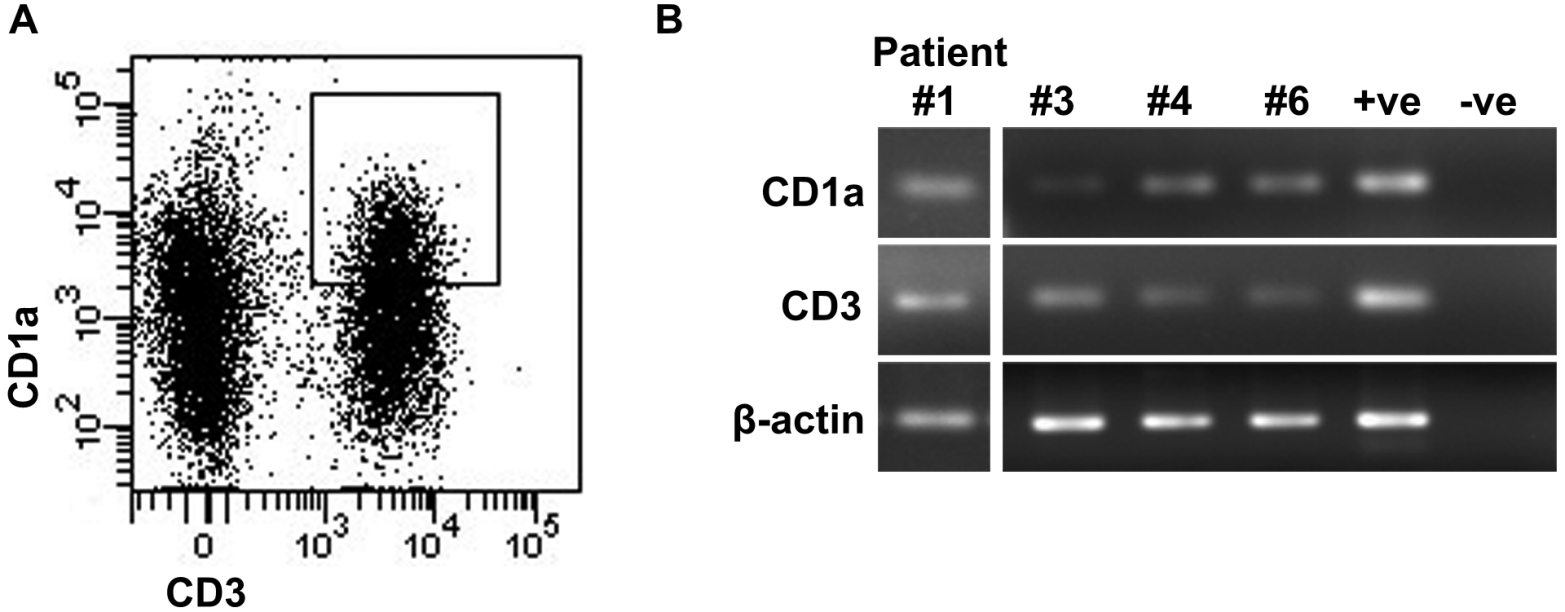
Expression of CD1a and CD3 mRNA in CD1a^+^/CD3^+^ cells sorted by FACS from LCH lesions. (A) Plot showing anti-CD1a-FITC [NA 1/34] and anti-CD3-APC-H7 [SK7] staining of LCH lesional cells from patient #1 by flow cytometry. The boxed area shows the gate used to sort CD1a^+^/CD3^+^ cells. This plot is representative for all patients examined. RNA was extracted from the CD1a^+^/CD3^+^ sorted cells and reverse transcribed for PCR amplification. (B) Agarose gel electrophoresis of RT-PCR products generated using primers specific for CD1a, CD3 and β-actin. PCR was initially performed with sorted cells from patient #1 and was later performed with sorted cells from patients #3, #4 and #6. PCR was not possible on sorted cells from patients #2 and #5. The positive control (+ve) was a cDNA sample previously shown to contain the amplicon and the negative control (-ve) was a reaction with no cDNA added.

PCR amplification of CD1a and CD3 confirmed our hypothesis that the CD1a^+^/CD3^+^ sorted cells from LCH lesions expressed both CD1a and CD3 mRNA in all four LCH lesions examined (Figure 6). β-actin could not be successfully amplified in the remaining two lesions, hence they were excluded. Amplification of CD1a from patient #3 was successful but faint. Using cDNA from CD1a^+^/CD3^+^ sorted cells; CD1a was successfully amplified from all four LCH lesions with a second pair of CD1a primers directed towards a different region of the CD1a sequence (data not shown).

Amplified CD1a mRNA from CD1a^+^/CD3^+^ sorted cells from patient #1 was sequenced. This was performed to confirm that the bands obtained were true CD1a sequence and that the CD1a transcript matched the reference sequence. Normal CD1a sequence was obtained over 1035 nucleotides from position 787-1822 when compared to the 2111 bp NCBI reference sequence (NM_001763.2 GI:110618223). This included 78.5% (728/927) of the mature peptide sequence.

## Discussion

Here we demonstrate for the first time, to our knowledge, the presence of CD1a^+^ T-cells in all LCH lesions we have studied to date. These cells were not detected in the peripheral blood from LCH patients. Cellular morphology was typical of lymphocytes, and these cells expressed many of the typical T-cell markers. We have shown that sorted CD1a^+^/CD3^+^ cells from LCH lesions also expressed both CD1a and CD3 mRNA, indicating that the CD1a was generated within these cells and not transferred as protein by T-cell interaction with LCs.

Previous studies have confirmed that T-cells within LCH lesions are polyclonal [15], [18]. Our data also demonstrates that CD1a^+^ T-cells are polyclonal, and are comprised of a wide range of T-cell subtypes. Furthermore, a number of studies have used flow cytometry to identify cells in LCH lesions; these investigations were limited to two or three parameters and predominantly focused on pathogenic LCs [34]–[36].

Multi-parameter flow cytometry and cell sorting have aided our capacity to more precisely determine cell phenotypes within LCH lesions. The heterogeneous populations of cells identified in this study clearly show that the cellular composition in LCH lesions is more complex in regards to CD1a expression than was previously recognized [14], [34].

More recent work using cell-specific gene expression profiling compared T-cells from LCH lesions to those of peripheral blood of LCH patients [6]. Although not commented on by the authors, examination of the supplemental data revealed a 48.95-fold increase in CD1a expression in lesional T-cells. These data appear to support our observation of a distinct population of CD1a^+^ T-cells within LCH lesions.

It is evident from our data that CD1a expression has been induced across a range of T-cell subsets in LCH lesions. The co-expression of T-cell markers and the lack of DC marker expression suggests it is unlikely that CD1a^+^ T-cells originate from pathogenic LCs. In light of these data, the question must be posed as to how and why this might occur. Peripheral T-cells do not normally express CD1a; however CD1a expression on cortical thymocytes is well documented [27]. During thymocyte maturation, CD1a is down-regulated so it is not normally expressed by T-cells in the peripheral circulation [27], [38]. Since our studies show that CD1a^+^ T-cells were not detected in the peripheral blood from LCH patients, it is unlikely that these cells represent circulating immature cortical thymocytes.

T-cells expressing CD1a were recently identified in very low numbers in the lymphoid tissue of tonsils [33]. Although we examined tonsil tissues we only analyzed the epithelial layer, and found no CD1a^+^ T-cells. The only other previously reported occurrences of CD1a expression by T-cells is in some hematological malignancies such as precursor T-cell lymphoblastic leukemias and lymphomas [29]–[31]. This does not explain the presence of CD1a^+^ T-cells in LCH lesions, as further analysis of these cells detected both T_H_ and T_C_ cells, as well as various stages of T-cell differentiation. Combined with our observation of multiple TCR Vβ subsets within the CD1a^+^ T-cell population, we infer that these cells could not have arisen from a single neoplastic cell.

CD1a expression can be induced by various cytokine combinations *in vitro*. These studies demonstrated that hemopoietic myeloid progenitor cells treated with M-CSF, IL-3 and TGF-β1 *in vitro* could express CD1a as well as CD207 and E-cadherin [25]. Studies have demonstrated that a subset of CD34^+^ progenitor cells proliferated *in vitro* in the presence of GM-CSF and TNFα, to produce CD1a^+^ cells [39], and that the lipid micro-environment can modulate CD1a expression and differentiation of monocyte-derived DCs [26]. Little is known, however, regarding the modulation of CD1a expression on lymphoid cells. Although PHA-stimulated normal T-cells have demonstrated intra-cellular expression of CD1a [40], there is no expression of CD1a molecules on the cell surface. Cultures of both AML and acute lymphoblastic leukemia blasts *in vitro* with various cytokine preparations have led to the expression of CD1a on the blast cells [41].

It is known that the LCH micro-environment contains numerous cytokines including GM-CSF and TNFα [19], [42], [43], and taken into conjunction with the above studies it would be tempting to postulate that the cytokine storm within LCH lesions may be responsible for inducing CD1a expression in T-cells. Against this hypothesis is the observation, to the best of our knowledge, that similar inflammatory conditions such as familial hemophagocytic lymphohistiocytosis, rheumatoid arthritis and inflammatory bowel disease are not characterized by a predominance of CD1a^+^ cells. CD1a expression on cells that would not normally express CD1a appears to be unique to LCH and not evident in any other inflammatory conditions associated with cytokine storms.

Our observation of CD1a^+^ T-cells in LCH lesions may open up further opportunities for functional studies. Experiments to determine function were beyond the scope of this study. Due to the limited availability of live cells for analysis, any potential mechanisms must remain theoretical. It has been reported that pathogenic LCs in LCH poorly present alloantigens [24], [34] and therefore have a defect in antigen presentation. This was corrected by the addition of CD40L *in vitro* [34] indicating a possible *in vivo* defect in this area that could be examined further in future studies.

Debate around the classification of LCH as a neoplastic disease or immune dysfunction has been ongoing [6], [13]–[16], [18], [44]–[47]. Numerous investigators have suggested an immunological dysfunction due to the altered expression of cytokines [42], [43], [48], [49]. Alternatively, support for LCH as a neoplasm is based on publications that have shown clonality of the CD1a^+^ cells in LCH using the human androgen receptor assay and *BRAF* mutation studies [14]–[17], [45], [50]. Our studies indicate that the CD1a^+^ cells in LCH are comprised of pathogenic LCs and polyclonal T-cells and as such, cast doubt on the concept that all CD1a^+^ cells in LCH are clonal [14], [15]. Our results do not discount that pathogenic LCs are clonal. If anything should be taken from this long standing debate, it is that LCH is an unusual disease where perhaps immune dysfunction and neoplasia are interconnected.

We demonstrate that CD1a is not a unique marker for pathogenic LCs in LCH and a new definition to include CD3^−^ and CD1a^+^ and/or CD207^+^ cells or the presence of Birbeck granules is required for the identification of pathogenic LCs in LCH. We hypothesize, that defective cycling of CD1a molecules to the surface of pathogenic LCs may lead to redundancy within the immune system, whereby T-cells can be induced to express CD1a and present antigen within LCH lesions. In summary, our studies have identified for the first time, to our knowledge, the presence of polyclonal CD1a^+^ T-cells in LCH lesions and the presence of these cells is specific to LCH lesions.

## Materials and Methods

### Ethics statement

This research was approved by the Ballarat Health Services and St John of God Hospital Ballarat Human Research Ethics Committee, University of Ballarat (now Federation University Australia) Human Research Ethics Committee, and Karolinska Institutet Biobank. Informed consent was written.

### Patient samples

We obtained peripheral blood from nine LCH patients and lesional tissue samples from six LCH patients. Four LCH patients provided both peripheral blood and tissue (Table 1). Non-LCH control tissues were collected including five tonsils, one normal lymph node (non-involved), peripheral blood from six healthy volunteers, two AML samples, one chronic lymphoid leukemia and one T-cell lymphoma sample (Table 4).

### Sample preparation

Samples were prepared from fresh tissue then stored in liquid nitrogen in 10% DMSO (v/v) or used fresh. LCH lesions, except the dermal sample from patient #3, were prepared as SCS in media (IMDM (Gibco) supplemented with 2 mM L-glutamine, 50 µg/ml kanamycin (Invitrogen) and 10% (v/v) heat-inactivated fetal calf serum (Gibco)). The dermal sample was prepared from a skin biopsy of an LCH lesion as previously described ^[51]^ except cells were resuspended in media. Tonsil epithelium was prepared by removing thin strips from the external surface. The central lymphoid tissue was discarded. The epithelial strips were incubated for 45 min at 37°C in 5 ml of 10 mg/ml dispase II (Roche) prior to the removal of any remaining lymphoid tissue. The strips were further incubated in 4 ml of 0.3% trypsin for 10 min at 37°C then added to 4 ml of media. The suspension was filtered then incubated for 10 min at 37°C with 1 mg/ml DNAse-1. Following centrifugation, pelleted cells were resuspended in media. White blood cells were isolated from peripheral blood from both normal and diseased samples. Red blood cell lysis buffer (155 mM NH_4_Cl; 10 mM KHCO_3_ and 0.1 mM ethylenediaminetetraacetic acid) was added to peripheral blood, incubated at room temperature for 10 min, re-centrifuged then washed twice by resuspending the pellet in phosphate buffered saline pH 7.4.

### Immunocytochemistry

Cytospins of SCS were fixed with methanol. The DakoCytomation EnVision Doublestain system was used for chromogenic immunocytochemistry. For fluorescent immunocytochemistry, cytospins were blocked with Image-iT FX, incubated with primary antibodies (anti-CD3 [polyclonal], Dako; anti-CD1a [O10], Abcam), washed, labeled with secondary antibodies (Alexa Fluor 488 and Alexa Fluor 596, Invitrogen), washed and mounted with ProLong Gold antifade reagent with DAPI (4,6 diamidino-2-phenylindole) (Invitrogen Molecular Probes). Isotype-matched antibodies were used as negative controls.

### Flow cytometry analysis and cell sorting

**Figure 7:**
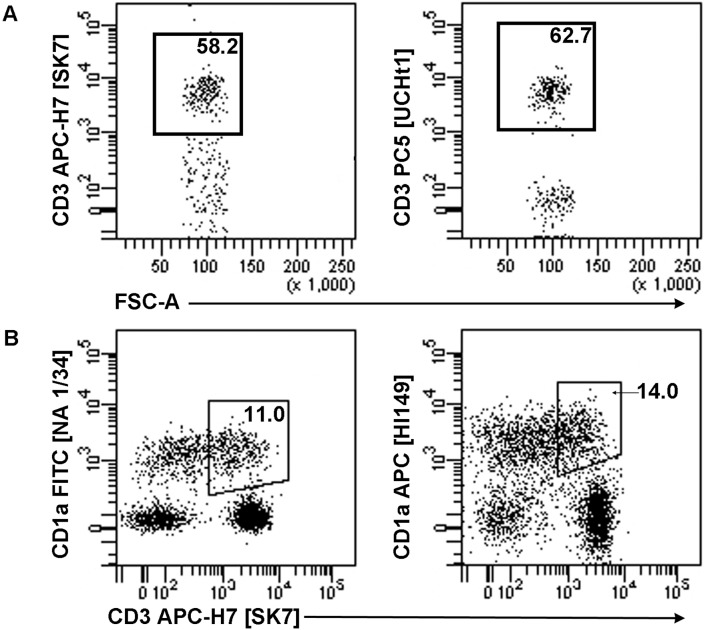
Comparisons of antibody binding specificities. (A) We used anti-CD3-APC-H7 [SK7] in all FACS analyses except for Vβ repertoire analyses, where anti-CD3-PC5 [UCHT1] was used as per IOTest Beta Mark TCR Vβ Repertoire Kit (Beckman Coulter) recommendation. A comparison between anti-CD3-APC-H7 [SK7] (left) and anti-CD3-PC5 [UCHT1] (right) using peripheral blood demonstrates that antibodies have a similar specificity. Plots show 100,000 events and frequency is expressed as a percentage of live lymphocytes. (B) Anti-CD3-APC-H7 [SK7] was used in combination with two different anti-CD1a antibodies for all FACS analyses excluding Vβ repertoire analyses. A comparison between anti-CD1a-FITC [NA 1/34] (left) and anti-CD1a-APC [HI149] (right) using a mix of peripheral blood and Jurkat cells shows that these antibodies have a similar specificity. Plots show 10,000 events and frequency is expressed as a percentage of live cells.

LCH biopsy specimens and controls were examined (Table 2, 4). The same cytometer settings were applied for all experimental samples to allow for direct comparison. Compensation controls, including single antibody stains and fluorescence minus one controls, were used to determine background and eliminate positive outliers. Prior to sample analysis, we compared antibody binding specificities to validate the use of more than one antibody-fluorophore combination for both anti-CD1a and anti-CD3 antibodies (Figure 7). Analysis of samples included dead cell gating by propidium iodide and the exclusion of doublets by forward/side scatter gating (Figure 2). Cells were analyzed with two scatter and eight fluorescence parameters and sorted into populations using a BD FACSAria II (BD Biosciences) and BD FACSDiva V6.1 software (BD Biosciences).

SCS were washed and resuspended in media. The cells were incubated with antibodies in the dark for 30 min at 4°C then washed twice. For phenotypic characterization, the following antibodies were used: anti-CD1a-FITC [NA 1/34], anti-CD1a-APC [HI149], anti-CD3-APC-H7 [SK7], anti-CD3-PC5 [UCHT1], anti-CD4-V450 [RPA-T4], anti-CD8-APC [SK1], anti-CD8-PE [HIT8a], anti-CD14-V450 [MφP9], anti-CD16-PE [3G8], anti-CD19-PE-Cy7 [SJ25C1], anti-CD20-PerCP-Cy5.5 [L27], anti-CD25-PE-Cy7 [M-A251], anti-CD31-AlexaFluor 647 [M89D3], anti-CD34-PE-Cy7 [8G12], anti-CD45-PerCP-Cy5.5 [2D1], anti-CD45RA-PE-Cy7 [HI100], anti-CD45RO-PE [UHCL-1], anti-CD56-PE-Cy5.5 [MEM-188], anti-CD123-PerCP-Cy5.5 [7G3], anti-CD138-PE [DL-101], anti-CD207-PE [DCGM4] and anti-CD303-FITC [AC144]. All antibodies were purchased from BD Biosciences except anti-CD1a-FITC (Dako), anti-CD56-PE-Cy5.5 (Invitrogen), anti-CD3-PC5 and anti-CD207-PE (Beckman Coulter) and anti-CD303-FITC (Miltenyi Biotec).

T-cells were analyzed using the IOTest Beta Mark TCR Vβ Repertoire Kit (Beckman Coulter). Analysis was performed according to recommendations (staining with anti-CD3-PC5 [UCHT1]) but with the addition of anti-CD1a-APC [HI149] (BD Biosciences).

### RNA isolation and reverse transcription-PCR

Total RNA was isolated using a miRNeasy Mini kit (QIAGEN) according to manufacturer’s instructions including the optional on-column DNase digestion. Random hexamer primers were used to synthesize cDNA using the Transcriptor First Strand cDNA synthesis Kit (Roche). PCRs were performed using 0.5–5 µl cDNA with gene-specific primers (Table 7) from PrimerBank (final concentration 0.4 µM) [52]–[54]. Final concentration of β-actin primers [55] was 0.125 µM. All primer pairs were intron spanning. Reactions were performed using 1 unit of AmpliTaq Gold DNA polymerase with GeneAmp PCR Gold Buffer, 1 mM MgCl_2_ (Applied Biosystems) and a final concentration of 200 µM for each dNTP (Roche). Thermal cycling was carried out on either an Applied Biosytems GeneAmp PCR system 2700 or a Perkin Elmer GeneAmp PCR system 2400 machine. Reactions were preheated at 95°C for 7 min followed by 40 cycles (95°C for 30 sec, 55°C for 30 sec, 72°C for 30 sec), with a final extension at 72°C for 10 min. As previously published, the annealing temperature used for β-actin reactions was 68°C [55]. Reactions were performed under hot start conditions and a positive control (sample previously shown to contain the amplicon) and negative control (no template) were included with every set of reactions. PCR products were separated on an agarose gel, stained with ethidium bromide, illuminated on an ultraviolet light box and photographed using a digital camera.

**Table 7:** Primer sequences for reverse transcription-PCR.

GeneName	PrimerBank ID#	Forward PrimerSequence (5′ to 3′)	Reverse Primer Sequence(5′ to 3′)	AmpliconSize (bp)
CD1a pair 1	27764865a1	GTGATGGCAATGCAGACGG	CCACAGGAAAACGATGGTGCT	164
CD1a pair 2	27764865a2	GTCCAGGGGAAACTTCAGCAA	CCTGTCACCTGTATCTCAAAAGG	141
CD3	4502671a1	TTTGGGGGCAAGATGGTAATG	GGGGTAGCAGACATAATAACCAC	245
β-actin	Not Applicable*	GGCGGCACCACCATGTACCCT	AGGGGCCGGACTCGTCATACT	202

# Wang and Seed B (2003) Nucleic Acids Res 31(24): e154.

Spandidos et al. (2008) BMC Genomics 9: 633.

Spandidos et al. (2010) Nucleic Acids Res 38(Database issue): D792–D799.

* Abrahamsen et al. (2003) J Molecular Diagnostics 5(1): 34–41.

### Sequencing CD1a

CD1a was sequenced from 3′-RACE reactions using RNA from CD1a^+^/CD3^+^ sorted cells from LCH patient #1 and the CD1a pair 1 forward primer (Table 7). SMART RACE cDNA Amplification Kit (Clontech) was used (following manufacturers’ instructions) with an annealing temperature of 60°C and 35 amplification cycles. A semi-nested reaction was performed using the CD1a pair 2 forward primer (Table 7) with an annealing temperature of 62°C and a further 30 amplification cycles. The 3′-RACE product was gel purified (QIAquick gel extraction kit, QIAGEN) and the PCR product (80 ng) directly sequenced using the standard cycle sequence protocol with the CD1a pair 2 forward primer (Micromon Services, Monash University).

### Statistical analysis

Data was analyzed in the GraphPad Prism statistical program (GraphPad Software, San Diego, CA). Non-parametric tests were used due to non-normality of small sample sizes. A Kruskal-Wallis one-way analysis of variance was used to detect differences between the percentage of CD1a^+^/CD3^+^ cells in LCH lesions, LCH peripheral blood and control tissues. While two LCH patients provided lesional tissue only, and five provided peripheral blood tissue only, four LCH patients provided both lesional and peripheral blood tissues. Being less powerful than a paired test to detect differences, an independent test was the most suitable option despite some pairing. Two-tailed Mann-Whitney U tests were conducted to detect differences between the percentage of T_H_ cells in the CD1a^+^/CD3^+^ and the CD1a^−/^CD3^+^ populations, and between the percentage of T_C_ cells in the CD1a^+^/CD3^+^ and the CD1a^−/^CD3^+^ populations. Data are quoted as means ± standard deviation. Differences were considered statistically significant at an alpha level of 0.05.
